# Personalized musculoskeletal modeling for gait analysis and decision-making in femoral derotational osteotomy for children with cerebral palsy

**DOI:** 10.1186/s13018-025-06287-w

**Published:** 2025-09-26

**Authors:** Jehyun Yoo, Kun-Bo Park, Juntaek Hong, Junmin Cha, Jeuhee Lee, Yebin Cho, Dong-wook Rha

**Affiliations:** 1 Department of Rehabilitation Medicine, Gachon University Gil Medical Center, Incheon, Republic of Korea; 2 Division of Pediatric Orthopedic Surgery, Severance Children’s Hospital, Yonsei University College of Medicine, Seoul, Republic of Korea; 3 Department and Research Institute of Rehabilitation Medicine, Yonsei University College of Medicine, 50-1, Yonsei-ro, Seodaemun-gu, Seoul, Republic of Korea

**Keywords:** Cerebral palsy, Femoral derotational osteotomy, Gait analysis, Hip internal rotation, Musculoskeletal modeling

## Abstract

**Background:**

Preoperative gait analysis plays a crucial role in determining the necessity and correction angle for femoral derotational osteotomy (FDO). However, conventional musculoskeletal models used in gait analysis often fail to reflect patient-specific musculoskeletal characteristics, such as femoral and tibial deformities. This study evaluates the impact of a personalized musculoskeletal model incorporating these deformities on gait analysis and investigates the surgical outcomes of FDO based on kinematic changes derived from this personalized musculoskeletal model.

**Methods:**

A retrospective analysis was conducted on 254 limbs from 127 children with cerebral palsy (CP) who presented with increased femoral anteversion and underwent pre- and postoperative gait analyses. Kinematic data were generated using general and personalized musculoskeletal models developed in OpenSim. Patients were classified according to FDO status and the presence of excessive hip internal rotation (IR). Surgical outcomes were assessed based on postoperative changes in hip rotation. Subgroup analyses were performed to evaluate the model’s impact on surgical outcomes.

**Results:**

Of the 254 limbs, 92 underwent FDO. Patients with increased hip IR in the general model (Group A) had a higher good responder rate (88.2%) than those without (Group B, 17.2%). All limbs in Groups A1 and B1 (increased hip IR using personalized musculoskeletal models) had 100% favorable outcomes, whereas Groups A2 and B2 (not increased hip IR using personalized musculoskeletal models) showed favorable outcomes in 20% and 13.5%, respectively. Increased hip IR was more frequent in patients with external tibial rotation (*p* < 0.05). Surgical outcomes differed significantly between patients with and without increased hip IR in the personalized musculoskeletal model (χ^2^ = 4.90, *p* = 0.027).

**Conclusion:**

Gait analysis using personalized musculoskeletal models improved surgical decision-making for FDO, leading to better outcomes in children with CP. Personalized musculoskeletal models better identified suitable FDO candidates and more accurately predict surgical outcomes than general models.

*Trial registration*: Retrospectively registered.

**Supplementary Information:**

The online version contains supplementary material available at 10.1186/s13018-025-06287-w.

## Introduction

Cerebral palsy (CP) is associated with various musculoskeletal problems, such as muscle shortening and bony deformities, which limit the range of motion in joints and contribute to pathological gait patterns that compromise gait speed and balance [1, 2]. Surgical intervention is often required to address musculoskeletal deformities that result in functional impairment. Femoral derotational osteotomy (FDO) is typically performed to correct the increased hip internal rotation (IR) caused by femoral anteversion. The decision to perform femoral derotational osteotomy (FDO) is primarily guided by clinical examination and the presence of increased femoral anteversion, which can be accurately measured using computed tomography (CT) [3, 4]. Gait analysis also plays a crucial role in identifying the in-toe gait and increased hip IR during gait, serving as a critical factor in determining the need for surgical intervention [5]. Incorporating gait analysis results into the decision-making process for surgical candidacy is associated with improved patient outcomes [6]. Specifically, patients exhibiting hip IR greater than 15° during gait analysis may be considered suitable candidates for FDO [7].

Conventional gait analysis is performed by recording a patient with markers attached to anatomical landmarks using an infrared camera. Kinematic data are then obtained based on the marker coordinate data using a digital human model. However, as conventional gait analysis relies on a general digital human model, it may not accurately reflect the musculoskeletal deformities in children with CP [8–11].

Several studies have explored the effect of personalized musculoskeletal conditions on gait analysis to evaluate and plan therapeutic interventions for children with CP. One study highlighted the potential differences in the sagittal plane hip and knee kinematics and transverse plane hip kinematics when using a magnetic resonance imaging (MRI)-based model [10]. Furthermore, inverse kinematic analysis using a personalized model that accounts for excessive femoral anteversion and tibial external rotation (ER) showed increased hip IR than that indicated by a general model [9]. The general model underestimated the moment arm of the hip internal rotator compared with that measured using the MRI-based personalized model [8]. Moreover, contrary to the general model, the knee joint loading can be varied in a personalized model [11]. However, no study has evaluated the functional gait changes or surgical outcomes achieved using personalized models for gait analysis. When performing FDO, determining the degree of correction based on the femoral anteversion angle within the normal range on CT or the gait analysis results derived from a general musculoskeletal model may have inherent limitations, potentially leading to over-correction or under-correction. By contrast, gait analysis results obtained using a personalized musculoskeletal model may offer a more precise estimation of the optimal surgical correction specific to the individual. Therefore, it is necessary to develop and use a personalized model that accurately calculates the kinematic and kinetic values in gait analysis to determine appropriate therapeutic interventions and surgery [12].

This study evaluates the effect of a personalized model reflecting femoral and tibial deformities in gait analysis and subsequently analyzes the surgical outcomes of FDO based on kinematic changes using the personalized model. The remainder of this article is structured as follows. In Sect. 2, we describe how patients were categorized, how the personalized musculoskeletal model was constructed, and how kinematic data were obtained using this model. This section also outlines the criteria used to evaluate surgical outcomes. In Sect. 3, we present the detailed results. Sections 4 and 5 discuss our findings and study limitations, respectively. Finally, in Sect. 6, we summarize our findings and present the conclusion.

## Methods

This study included 254 legs of 127 ambulatory patients with CP—classified as gross motor function classification system levels I and II—who underwent orthopedic surgery for musculoskeletal deformities between January 1, 2014 and December 31, 2024, followed by both preoperative and postoperative gait analyses (Table 1). Inclusion criteria were limited to patients with a preoperative CT-measured femoral anteversion of 30° or more, indicating the need for evaluation or treatment [7, 13, 14]. Patients who underwent more than one surgery at different times or who received other interventions between gait analyses, such as botulinum toxin injections, were excluded. The electronic medical records were reviewed to verify the type of surgery the patients underwent and to determine whether an FDO was performed. The degree of correction for the FDO was determined by a surgeon with more than 20 years of experience, referencing both the CT findings and gait analysis results. In cases where FDO was performed unilaterally, a correction was made to approximate the angle of the contralateral side. When a distal soft tissue surgery was performed concurrently, the correction was slightly reduced compared to the target angle, considering the effects of the soft tissue procedure.

**Table 1:** Demographic information (*n* = 127)

Demographic variable	No. (%)
Sex	
Male	81 (63.77%)
Female	46 (36.22%)
Age (years)	
Range	10–24
Median	18 years
IQR	Q1: 15.0,
	Q3: 21.0
GMFCS	
Level 1	43 (33.85%)
Level 2	84 (66.14%)
FDO status	
Bilateral FDO	33 (25.98%)
Unilateral FDO (right)	11 (8.66%)
Unilateral FDO (left)	15 (11.81%)
No FDO	68 (53.55%)

*GMFCS* gross motor function classification system, *IQR* interquartile range, *FDO* femoral derotational osteotomy

**Fig. 1:**
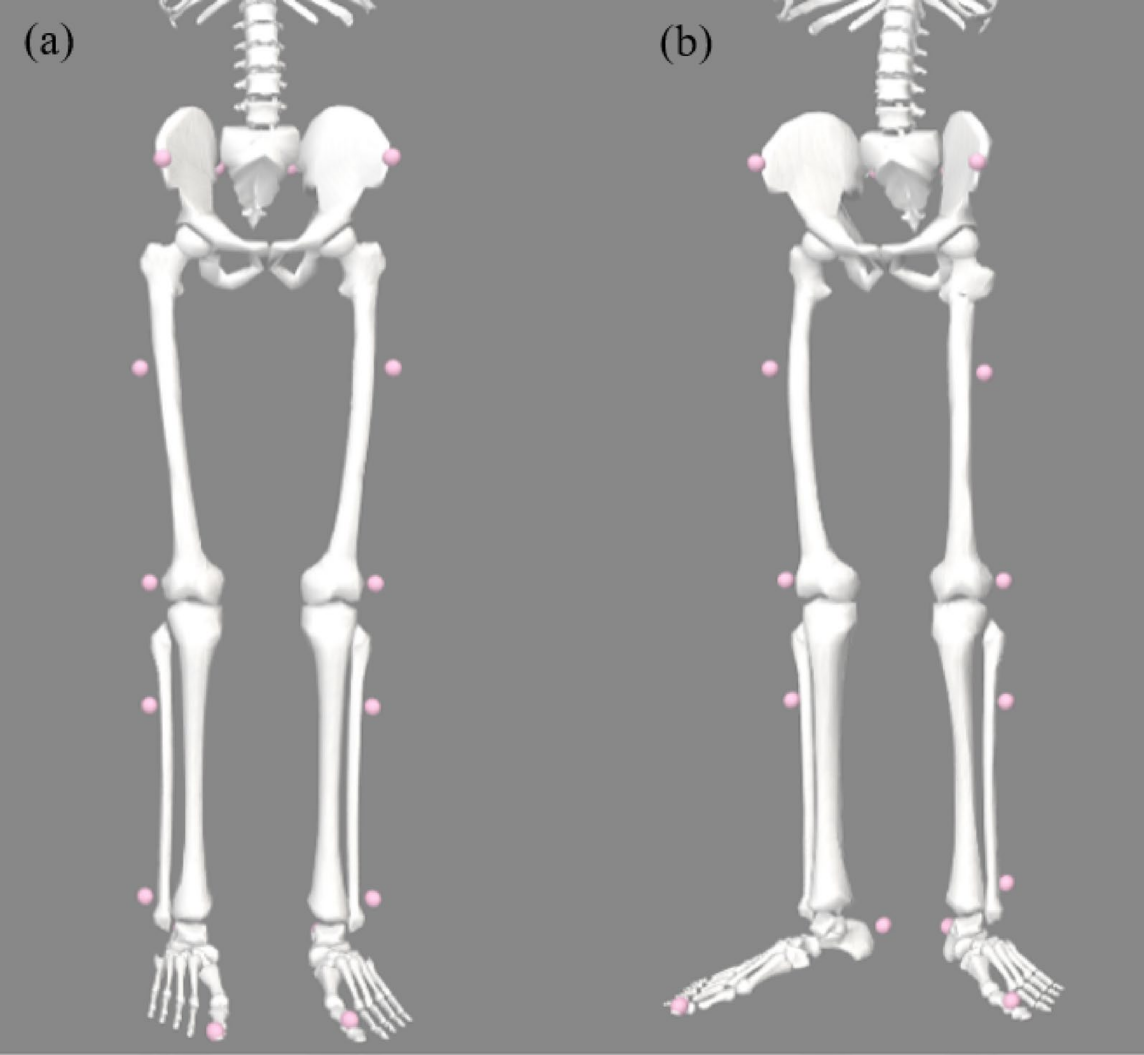
**a** General model (OpenSim 2392 model); **b** example of personalized model (Femoral anteversion: 38.1/32.1°, Neck shaft angle: 141.9/147.9°, and Tibial rotation: 37.4/36.6°)

OpenSim [15] was used to handle the musculoskeletal model and obtain the kinematic data based on the results of gait analysis. An OpenSim 2392 model with 23 degrees of freedom and 92 musculotendon actuators was used as the general musculoskeletal model. A personalized OpenSim model reflecting the patient’s skeletal deformities was created using the Torsion Tool (https://simtk.org/projects/torsiontool), an open-source software based on MATLAB (MathWorks, Natick, MA, USA). This personalized model was constructed using specific geometric parameters measured from preoperative lower extremity CT scans by a radiologist specializing in diagnostic imaging. These parameters—including femoral anteversion, femoral neck–shaft angle, and tibial external rotation—were quantified and incorporated into the model to accurately reflect the patient’s skeletal characteristics. Both models were scaled according to the patient’s height and weight (Fig. 1).

Pre- and postoperative gait analyses were performed using a marker-based motion capture system equipped with the VICON camera system (VICON MX-T10, Oxford Metrics Inc., Oxford, UK), employing the Helen Hayes marker set. During the analysis, children were instructed to walk as they typically do in daily life, in terms of both speed and gait pattern. The marker data obtained from the preoperative gait analysis were used to calculate kinematic data using the inverse kinematics tool in OpenSim, initially with a general model and subsequently with a personalized model. In the preoperative gait analysis, an increase of more than 15° in maximal hip IR during the stance phase was considered indicative of excessive hip IR [16]. Postoperatively, patients were classified as “good responders” if their maximal hip IR during the stance phase decreased by more than 15°, and the absolute hip rotation angle during gait fell within ± 15°. Conversely, patients who did not meet these criteria were classified as “non-responders” [17, 18].

Patients were classified based on whether they underwent FDO and whether increased hip IR was observed using the general model in the preoperative gait analysis. Patients who underwent FDO were classified into Group A (with increased preoperative hip IR) and Group B (without increased preoperative hip IR). The ratio of good responders to non-responders was calculated for each group.

The kinematic data were recalculated using a personalized model derived from the patient’s CT data. Group A was further divided into two subgroups: A1 and A2. A1 included patients who showed increased hip IR using the personalized model, whereas A2 included those who did not. Group B was further divided into two subgroups: B1 and B2. B1 included patients who showed increased hip IR using the personalized model, whereas B2 included patients who did not. The ratios of good responders to A1, A2, B1, and B2 were calculated.

**Fig. 2:**
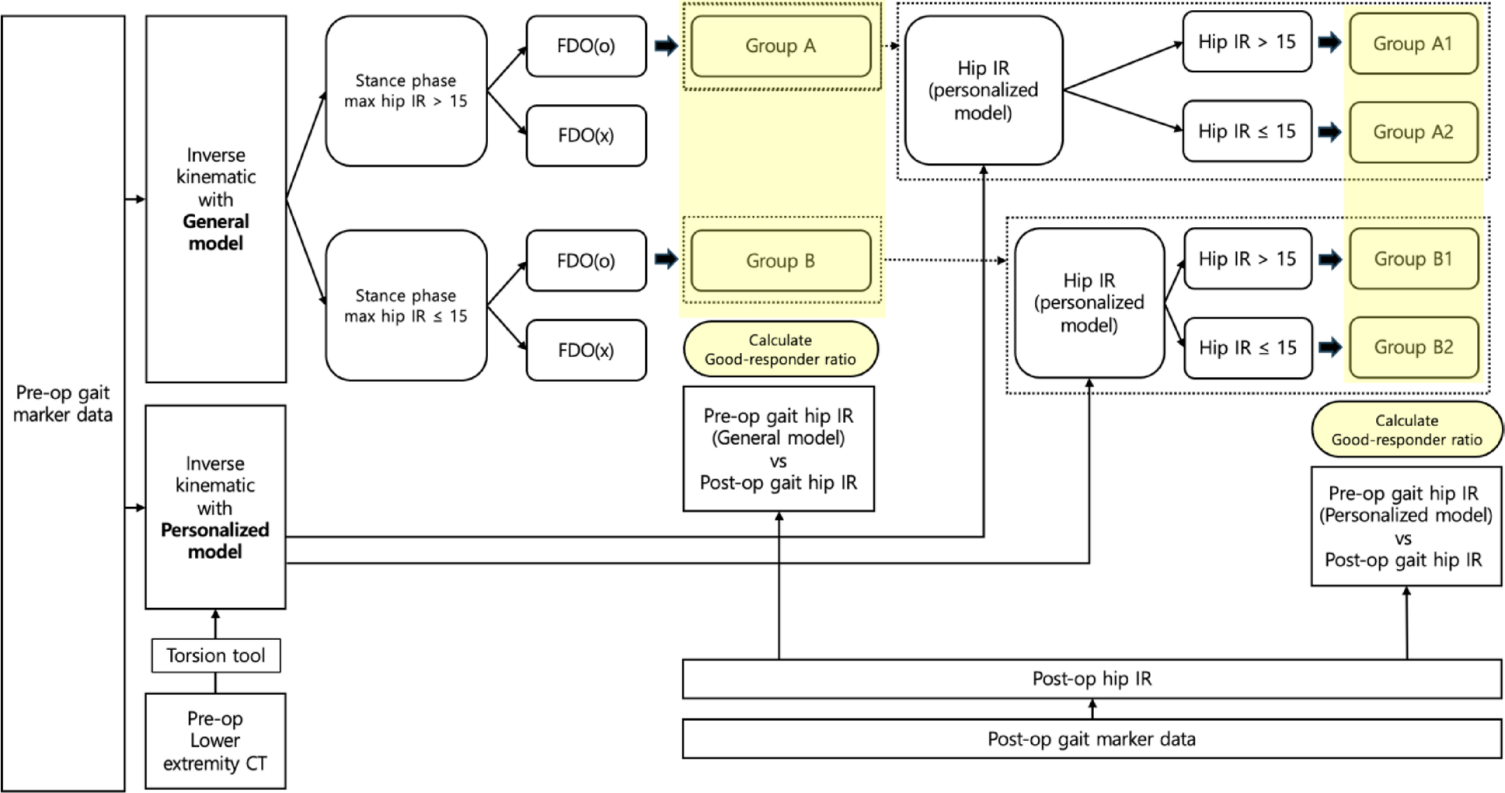
Schematic representation of the research process

The effect of the personalized model on the changes in hip IR was analyzed. The patients were divided into two groups based on the presence or absence of excessive tibial ER. Subsequently, the two groups were compared with respect to the changes in hip IR when the personalized model was applied using a paired *t* test, with *p* < 0.05 considered statistically significant. Figure 2 shows the schematic diagram of this study. This study was approved by the Institutional Review Board of Yonsei University (IRB approval number: 4-2023-1380).

## Results

**Fig. 3:**
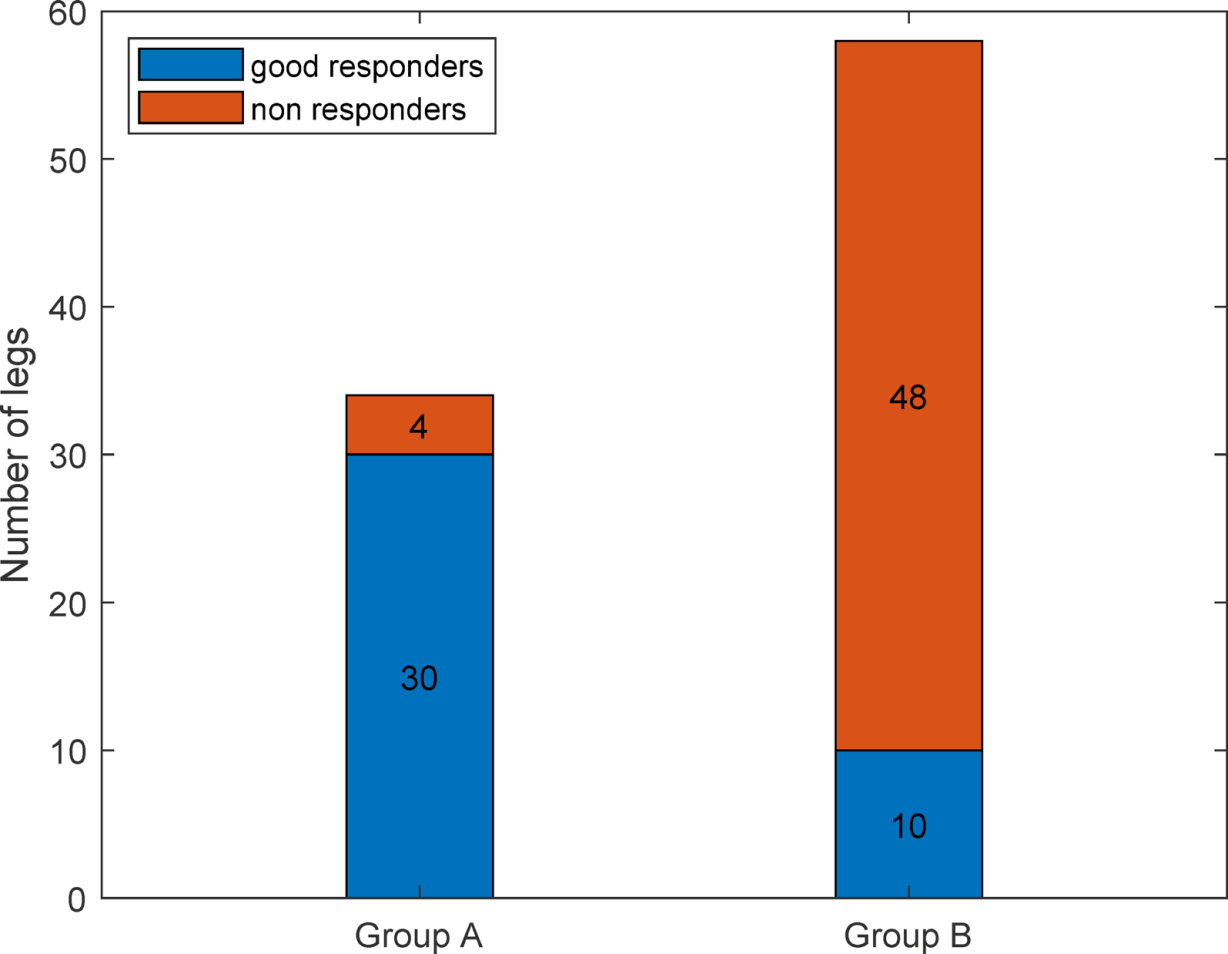
Number of good responders and non-responders based on the classified group—Group A: hip IR increased with the general model; and Group B: hip IR is not increased with the general model

A total of 254 legs of 127 patients were included, of whom 92 had undergone FDO. Group A (increased hip IR and FDO) included 34 legs with a good responder ratio of 88.23%. Group B (patients who did not show increased hip IR and underwent FDO) included 58 legs with a good responder rate of 17.24% (Fig. 3).

**Fig. 4:**
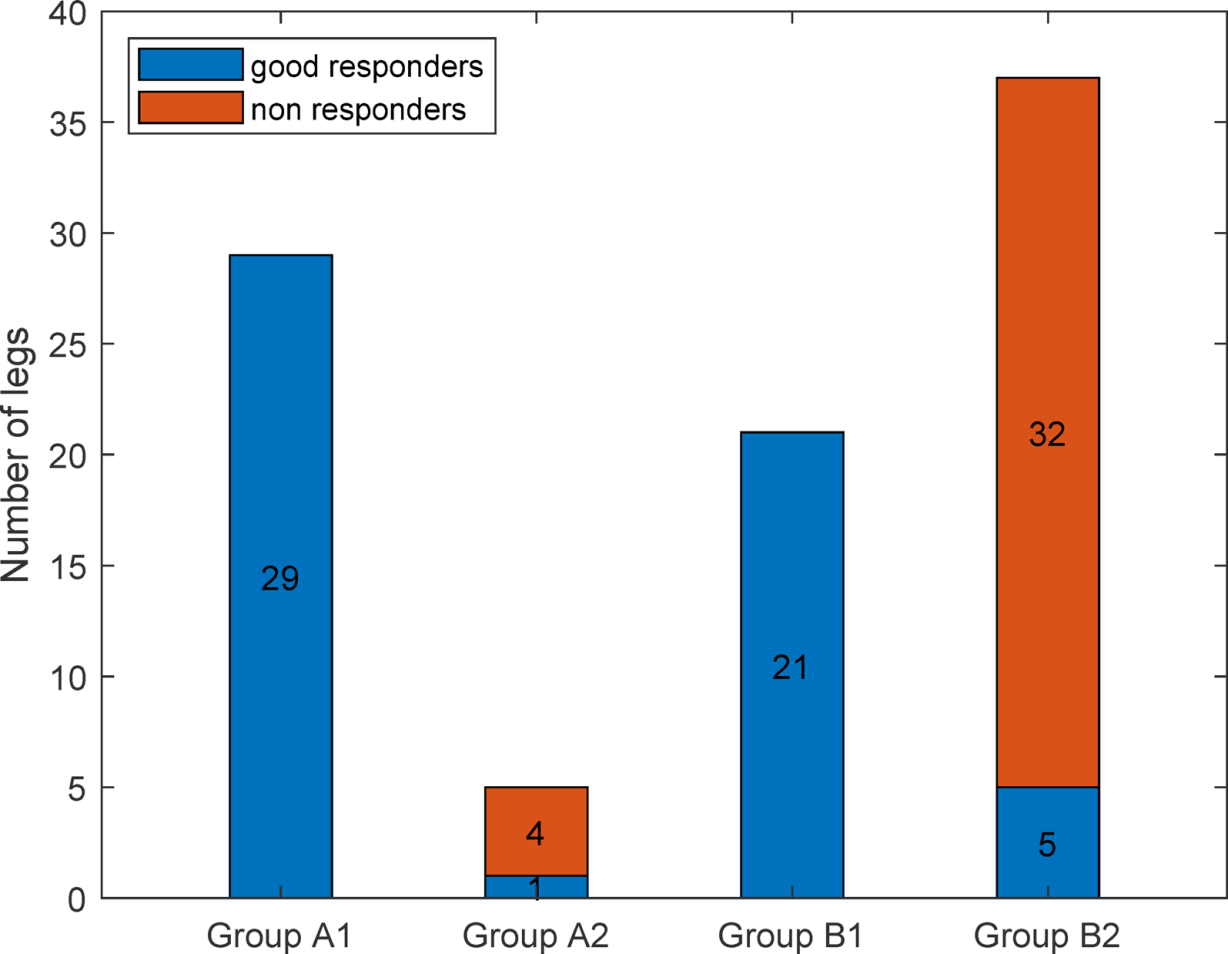
Surgical outcomes according to subgroups categorized based on the personalized model. Group A1: Hip IR increased with the personalized and general models; Group A2: Hip IR did not increase with the personalized model but increased with the general model; Group B1: Hip IR increased with the personalized model, but not with the general model; Group B2: Hip IR did not increase with the personalized model or the general model

**Fig. 5:**
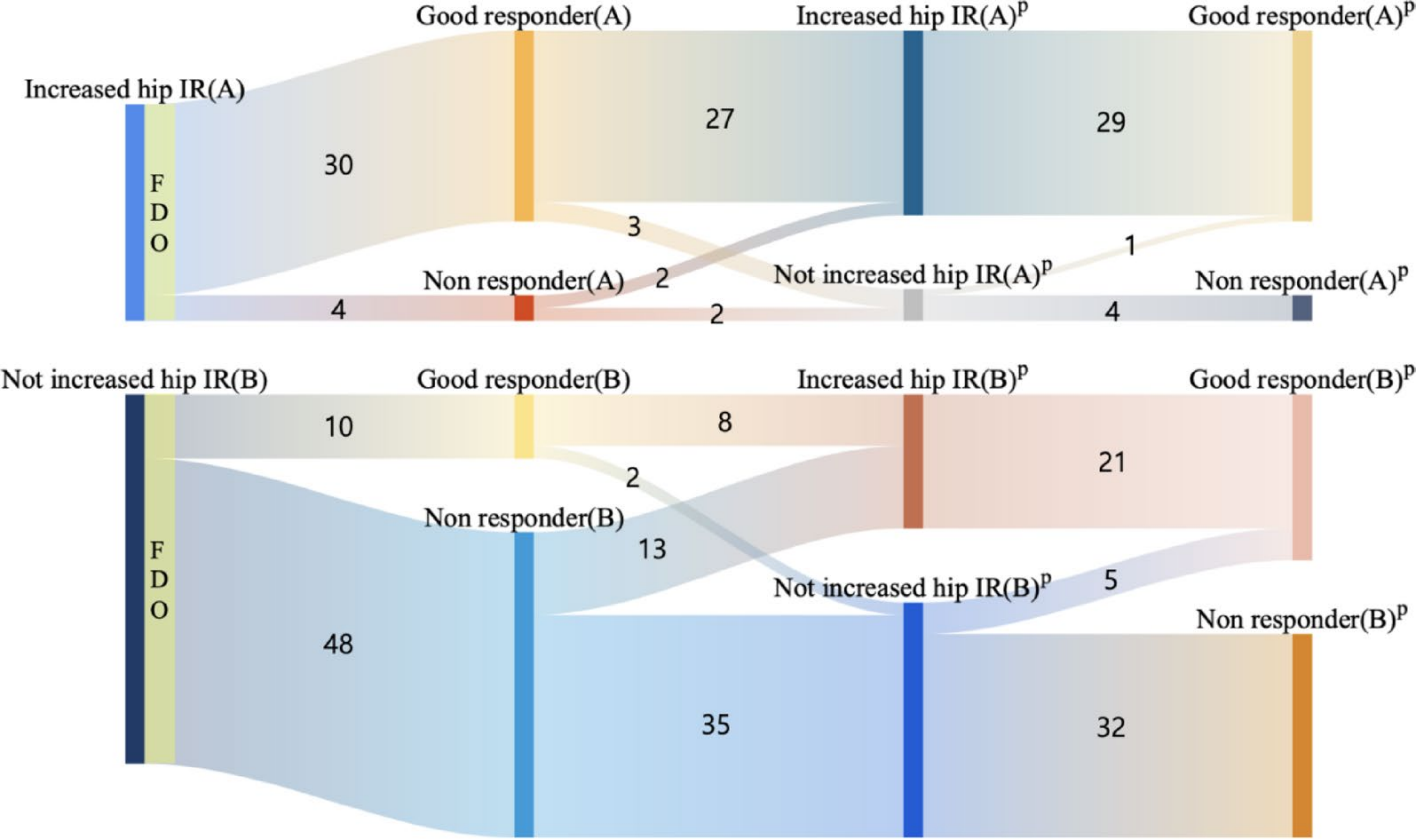
Sankey diagram of the surgical outcomes of FDO based on general and personalized models. ‘(p)’ indicates the kinematic values obtained using the personalized model. (FDO: Femoral derotational osteotomy, IR: internal rotation)

Group A1 (patients who showed increased hip IR in both the general and personalized models and underwent FDO) included 29 legs, all of which were good responders. Group A2 (patients who showed increased hip IR in the general model but not in the personalized model and underwent FDO) included five legs, with only one good responder. Group B1 (patients who did not show increased hip IR in the general model but did in the personalized model) included 21 legs, all of which were good responders. In contrast, Group B2 (patients who did not show increased hip IR in the general or personalized model) had a good responder rate of 13.51% (Fig. 4). Figure 5 illustrates the surgical outcomes of FDO using a Sankey diagram based on both general and personalized models.

**Fig. 6:**
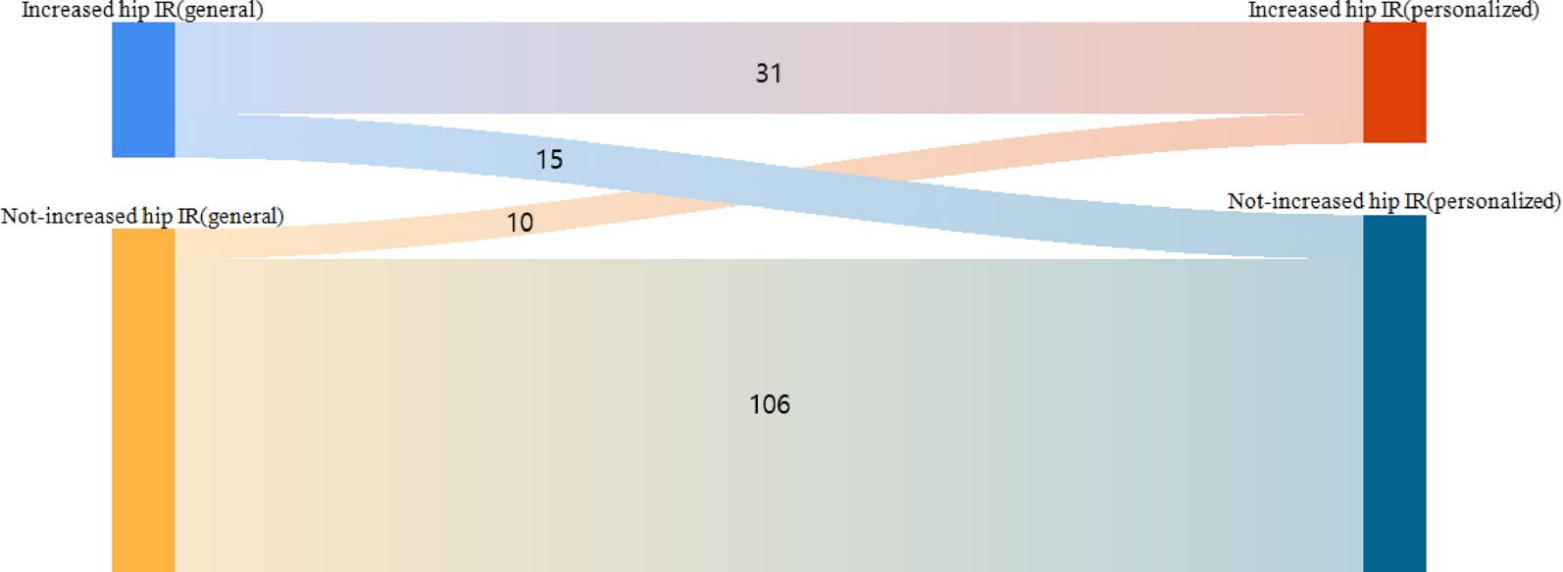
Sankey diagram of changes in hip IR derived from general and personalized models in the non-FDO group. (FDO: Femoral derotational osteotomy, IR: internal rotation)

A total of 162 limbs were included in the non-FDO group. Among these, 46 limbs exhibited increased hip IR when analyzed using the general musculoskeletal model, and 31 limbs continued to show increased hip IR in the personalized model. The remaining 116 limbs did not show increased hip IR in the general model; however, 10 demonstrated increased hip IR when evaluated using the personalized model (Fig. 6).

**Fig. 7:**
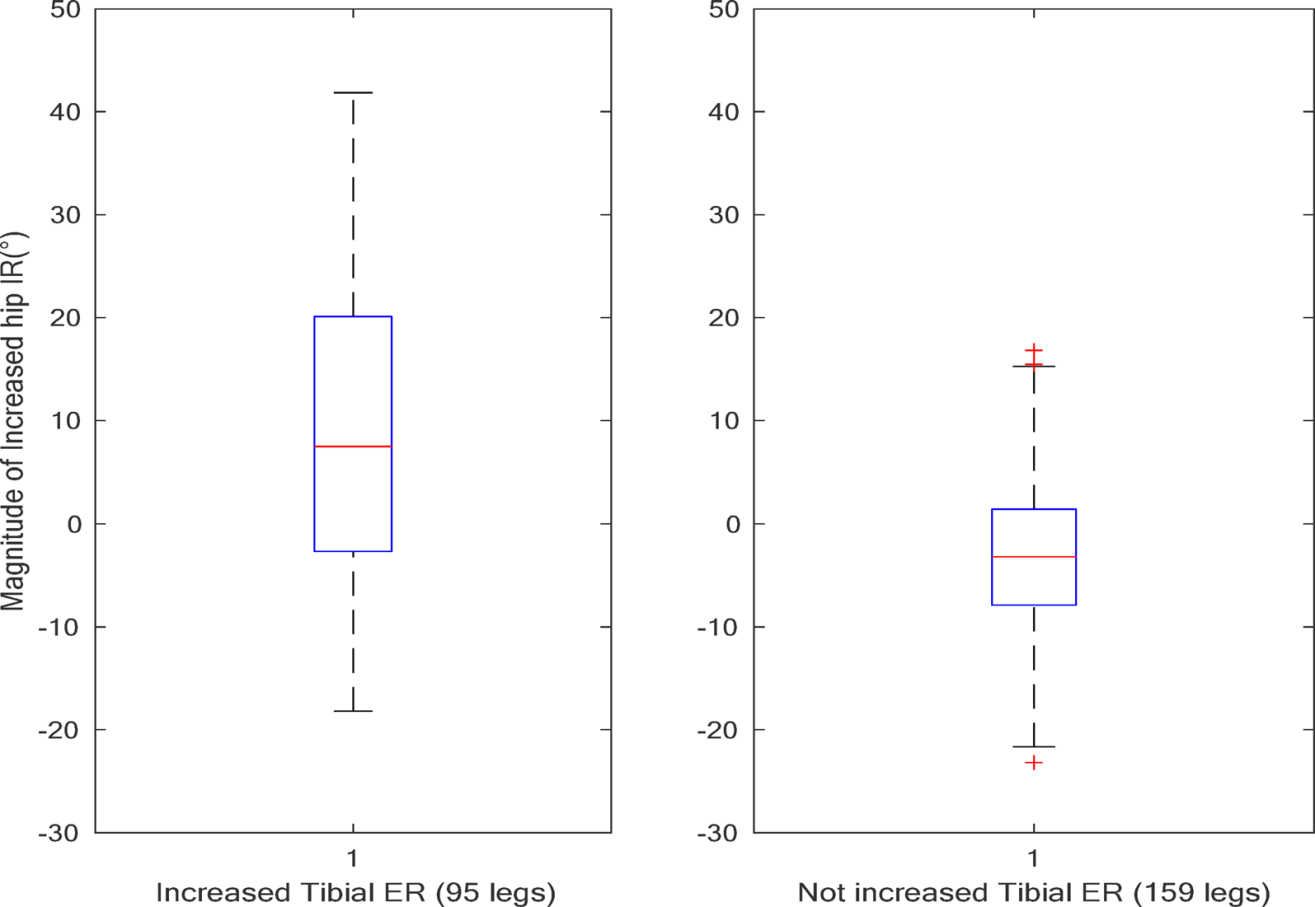
Effect of increased tibial ER on the maximal hip IR during the stance phase using a personalized model. (IR: internal rotation, ER: external rotation)

The difference in hip IR obtained using the general and personalized models was analyzed in relation to the tibial ER. In the group with increased tibial ER (95 legs), the application of the personalized model resulted in increased maximal hip IR during the stance phase compared to the group with non-increased tibial ER (159 legs) (*p* < 0.05) (Fig. 7).

The differences in the composition of good responders and non-responders based on the general and personalized models were compared using the chi-square test. A statistically significant difference was observed between the general and personalized models (Table 2) (χ^2^ = 4.90, *p* = 0.027).

**Table 2:** Differences in the surgical outcome classification between general and personalized musculoskeletal models

	Good responder (legs)	Non-responder (legs)
General model		
Hip IR increased (Group A)	30	4
Hip IR not increased (Group B)	10	48
Personalized model		
Hip IR increased (Group A1 + B1)	50	0
Hip IR not increased (Group A2 + B2)	6	36

## Discussion

This study analyzed the effect of a personalized musculoskeletal model in assessing the impact of FDO in ambulatory children with CP presenting with femoral anteversion on CT data. While several studies have previously demonstrated the importance of personalized models in gait analysis [8–10], this study evaluated the surgical outcomes of FDO using personalized gait analysis models.

When children with CP present with increased femoral anteversion, they tend to exhibit increased internal hip rotation and an in-toeing gait, which also affects pelvic tilt, maximal hip power generation and absorption, and knee power absorption [19]. Excessive femoral anteversion can damage the knee joint cartilage [20]. FDO is often considered to correct hip IR caused by excessive femoral anteversion and has shown good surgical outcomes in long-term follow-up [21].

To determine the need for FDO, several factors should be considered, including in-toeing gait, increased femoral anteversion on CT, increased femoral anteversion on physical examination, and 3D gait analysis [7]. The increased hip IR observed in the 3D gait analysis plays a crucial role in deciding whether to perform FDO. In most motion laboratories, a general model based on able-bodied individuals is used to derive joint kinematic values, even though patients with conditions such as CP often have different musculoskeletal characteristics, typically including femoral anteversion and external tibial rotation [2]. In this study, personalized models for children with CP were created by incorporating femoral anteversion, coxa valga, and tibial ER from preoperative CT data. Using these personalized musculoskeletal models, hip rotation was recalculated during preoperative gait analysis. Comparison of gait kinematics from general and personalized models using the same marker data showed that increased tibial ER led to greater hip IR in the kinematics generated by the personalized model (Fig. 5). This finding is supported by a previous study that reported that increased tibial ER can result in greater hip IR during the inverse kinematics process when certain musculoskeletal models are used to compute kinematics data [9].

In the preoperative gait analysis, patients who exhibited increased hip IR and underwent FDO (Group A) demonstrated a high good responder rate (30 of 34 limbs, 88.23%). When the personalized models were applied, 29 limbs in Group A still exhibited increased hip IR (Group A1), all of which showed favorable outcomes. In contrast, among the five limbs in which hip IR did not increase in the personalized model (Group A2), only one showed a favorable outcome after FDO. The remaining four limbs showed no variation in hip IR, but they demonstrated increased hip ER postoperatively, suggesting possible overcorrection due to surgery.

Patients who did not exhibit increased hip IR in the preoperative gait analysis but underwent surgery based on the clinician’s judgment (Group B) had a significantly lower good outcome rate when assessed using the general model (10 of 58 limbs, 17.24%). However, when gait analysis was re-evaluated using a personalized musculoskeletal model, 21 of the 58 limbs (36.2%) were found to exhibit increased hip IR preoperatively (Group B1), and all showed favorable outcomes. In contrast, among the remaining 37 limbs in which hip IR did not increase in the personalized model (Group B2), only five limbs were classified as good responders. The other 32 limbs showed no improvement in the hip IR or demonstrated increased hip ER, suggesting possible overcorrection.

These subgroup transitions and their corresponding surgical outcomes are illustrated in Fig. 5. Favorable outcomes were consistently observed in patients identified with increased hip IR by both models (A1, B1), whereas those classified as not having increased hip IR in the personalized model (A2, B2) demonstrated markedly lower success rates. Figure 6 shows the corresponding comparison in the non-FDO group, where the personalized model identified a small subset of limbs (8.6%) with increased hip IR that had been overlooked by the general model. This finding suggests that these patients might be appropriate surgical candidates if personalized gait analysis were incorporated into preoperative decision-making.

Surgeons do not rely solely on the results of the gait analysis to decide whether to perform FDO or determine the degree of correction. Rather, these decisions are made by integrating additional clinical information, such as CT data, physical examination findings, and the surgeon’s own experience. The observation that surgical outcomes improve when personalized models are used compared with general models suggests that preoperative gait analysis based on a general model may not adequately convey the patient’s actual biomechanical condition to the surgeon. Traditional gait analysis models used to capture kinematic data during patient ambulation are primarily based on normal musculoskeletal models. However, musculoskeletal deformities are highly prevalent in children with CP [1, 2], and using a general model that does not account for these deformities could distort kinematics data. There was a significant difference in surgical outcomes obtained using the general and personalized model, with the personalized model showing a higher proportion of good responders.

In Group A2, increased hip IR was observed in the preoperative gait analysis, indicating FDO. However, when re-evaluated using a personalized musculoskeletal model, no increase in hip IR was detected, suggesting that surgery might not be necessary in these cases. Notably, only one patient in this group achieved a favorable outcome, suggesting that reliance on general models may lead to suboptimal surgical decision-making in certain cases. Conversely, in Group B1, although the general gait analysis did not reveal increased hip IR, the surgeon, based on comprehensive clinical assessment and expertise, determined that femoral anteversion was the underlying cause of the gait abnormality and proceeded with FDO, resulting in favorable outcomes.

One hundred sixteen legs did not present with increased hip IR, and hence, did not undergo FDO. However, on evaluation using the personalized model, 10 legs (8.6%) demonstrated an increased hip IR. These patients are potential candidates for FDO surgery if the gait analysis using a personalized model is considered for decision making. If accurate information regarding hip IR are provided to the surgeon through preoperative gait analysis, these patients can be helped through timely surgical intervention. Therefore, it is vital to obtain kinematics data that reflect an individual’s unique musculoskeletal characteristics during gait analysis to administer appropriate treatment to patients who may benefit from surgical correction of gait.

The decision to perform FDO was influenced by multiple clinical factors beyond CT and gait analysis, and postoperative kinematics were derived from a general model rather than a personalized postoperative model which may have influenced the interpretation of the surgical outcomes. However, the findings of this study suggested that accurate gait analysis using a personalized musculoskeletal model before surgery may aid in determining the necessity and extent of surgical intervention, potentially leading to more favorable clinical outcomes.

The replication pipeline involves constructing a personalized musculoskeletal model from CT scan data to incorporate bone deformities, performing gait analysis with a marker-based motion capture system, and processing the acquired marker data through inverse kinematics in a simulation tool (e.g., OpenSim) to derive joint angles. The substantial financial and temporal costs associated with the current methodology present a barrier to its direct clinical implementation. Future prospective studies are needed to provide evidence that personalized motion analysis can influence surgical outcomes. If this evidence is established, it would justify the development of a semi-automated pipeline to improve feasibility and overcome current practical challenges.

## Limitations

This study had several limitations. The choice of surgical intervention for each patient may be influenced by various factors beyond CT data and gait analysis. These include findings of physical examination in the operating room after anesthesia, patient and guardian preferences, and the surgeon’s judgment based on clinical experience. However, owing to the retrospective nature of this study, a key limitation is the inability to account for such confounding factors. The personalized model only reflects femoral anteversion, neck-shaft angle, and tibial rotation. It does not consider other deformities like hip joint abnormalities, muscle shortening, or related conditions. Furthermore, as the study included patients who underwent more than one surgical procedure involving various bones and muscles of the lower limb, surgeries performed in regions other than the hip may have influenced the hip IR during gait. Although the personalized musculoskeletal model was generated using the patients’ preoperative CT data, postoperative analysis was conducted using a general model rather than a personalized postoperative model because the varying degrees of surgical correction could not be individually incorporated. This may have introduced bias in the interpretation of the surgical outcomes. This was a retrospective study, and the surgeons primarily relied on CT findings and gait analysis results obtained using a general model when determining the correction angle. Therefore, further prospective studies are needed to evaluate whether gait analysis using a personalized model can aid in determining the optimal angle of correction.

## Conclusions

To assess the need for FDO in children with CP who present with excessive femoral anteversion, a personalized model for gait analysis is proposed. By incorporating patient-specific femoral and tibial deformities, the personalized model provided a more accurate representation of gait kinematics, particularly hip IR—a key factor in determining the indication for FDO. Subgroup analyses revealed that favorable surgical outcomes were strongly associated with the presence of increased hip IR identified by the personalized model, whereas outcomes were less favorable when such abnormalities were absent. Integrating personalized gait analysis into the preoperative evaluation process may offer more precise information to surgeons, potentially leading to improved postoperative hip rotation outcomes. A prospective study comparing surgical outcomes for patients whose FDO was planned using gait analysis based on personalized versus general musculoskeletal models would be valuable for determining whether personalized modeling improves surgical appropriateness and outcomes.

## Supplementary Information

Below is the link to the electronic supplementary material.

Supplementary Material 1

## Data Availability

The data and materials are provided in the supplementary material.

## Abbreviations

CP: Cerebral palsy
CT: Computed tomography
FDO: Femoral derotational osteotomy
IR: Internal rotation
